# Comparative performance of artificial ıntelligence models in physical medicine and rehabilitation board-level questions

**DOI:** 10.1590/1806-9282.20240241

**Published:** 2024-07-19

**Authors:** Ahmet Kıvanç Menekşeoğlu, Enes Efe İş

**Affiliations:** 1 University of Health Sciences, Kanuni Sultan Süleyman Education and Training Hospital, Department of Physical Medicine and Rehabilitation – İstanbul, Turkey.; 2 University of Health Sciences, Sisli Etfal Education and Training Hospital, Department of Physical Medicine and Rehabilitation – İstanbul, Turkey.

**Keywords:** Artificial intelligence, Physical Medicine and Rehabilitation, Academic performance

## Abstract

**OBJECTİVES:**

The aim of this study was to compare the performance of artificial intelligence models ChatGPT-3.5, ChatGPT-4, and Google Bard in answering Physical Medicine and Rehabilitation board-style questions, assessing their capabilities in medical education and potential clinical applications.

**METHODS:**

A comparative cross-sectional study was conducted using the PMR100, an example question set for the American Board of Physical Medicine and Rehabilitation Part I exam, focusing on artificial intelligence models' ability to answer and categorize questions by difficulty. The study evaluated the artificial intelligence models and analyzed them for accuracy, reliability, and alignment with difficulty levels determined by physiatrists.

**RESULTS:**

ChatGPT-4 led with a 74% success rate, followed by Bard at 66%, and ChatGPT-3.5 at 63.8%. Bard showed remarkable answer consistency, altering responses in only 1% of cases. The difficulty assessment by ChatGPT models closely matched that of physiatrists. The study highlighted nuanced differences in artificial intelligence models' performance across various Physical Medicine and Rehabilitation subfields.

**CONCLUSION:**

The study illustrates the potential of artificial intelligence in medical education and clinical settings, with ChatGPT-4 showing a slight edge in performance. It emphasizes the importance of artificial intelligence as a supportive tool for physiatrists, despite the need for careful oversight of artificial intelligence-generated responses to ensure patient safety.

## INTRODUCTION

In the rapidly advancing domain of artificial intelligence (AI), various models such as ChatGPT-3.5, ChatGPT-4, and Google Bard have demonstrated notable proficiency in numerous academic studies, particularly within the context of medical examinations^1-3^. The integration of AI into clinical practices requires that these technologies not only comply with but also augment the procedural framework of medical professionals, with an emphasis on enhancing efficiency, accuracy, and reliability^4^. Consequently, evaluating these AI models' proficiency in interpreting and responding to specialized, board-style examination questions becomes a pivotal step in assessing their potential clinical utility. This research contributes to the scientific discourse by offering a detailed comparative analysis of these AI systems, specifically examining their relevance and efficacy in the specialized field of Physical Medicine and Rehabilitation (PMR), thereby laying the groundwork for future integration of AI in healthcare settings.

Physical Medicine and Rehabilitation is a discipline characterized by its holistic approach to patient care, necessitating an extensive understanding of a multifaceted treatment spectrum. The benchmark for our investigation is the PMR100, issued by the American Board of Physical Medicine and Rehabilitation (ABPMR). This compilation is reflective of the content scope and complexity inherent to the Part I Certification Examination in PMR^5^.

The primary objective of our study was to critically assess and compare the capabilities of ChatGPT-3.5, ChatGPT-4, and Google Bard in interpreting and responding to the intricate and specialized questions encompassed within the PMR100. Additionally, we aimed to examine the performance of these AI systems within various subfields of PMR, offering a comprehensive assessment of their proficiency and applicability across the spectrum of this discipline.

## METHODS

This was a comparative, cross-sectional study designed to evaluate and compare the performance of AI language models, specifically Bard and different versions of ChatGPT (3.5 and 4), in the context of PMR. The study aimed to assess the ability of these AI models to answer board-style questions and categorize them based on difficulty (easy, medium, and difficult). In this study, the AI models Bard (Google AI, Mountain View, CA, USA), ChatGPT-3.5 (OpenAI, L.L.C., CA, USA), and ChatGPT-4 (OpenAI, L.L.C., CA, USA) were used between January 20 and 25, 2024. Part I practice questions (PMR100) published by the ABPMR were used as a sample for the board exam. In the set of 100 questions, each question has one correct answer out of four options, and the answer key was provided by ABPMR. Out of a total of 100 questions, six were excluded from the study, and the data related to 94 questions were evaluated. Six questions were not evaluated because they contained videos or photographs.

Each AI model was presented with the questions, accompanied by a short introduction: "The following is a national board-level exam question for physiatrists. Read the question and indicate the level of difficulty as easy, medium, or difficult, then choose the correct option." After the first answer, each AI model was asked, "Are you sure?" to assess its confidence in the answer. Both answers and the level of difficulty were recorded. The answers were compared with the correct answer key provided by ABPMR.

The questions were also jointly graded by two European Board-certified physiatrists into three difficulty categories: easy, moderate, and difficult. Bloom's Taxonomy for Learning and Assessment Framework was employed to categorize the questions based on the necessary cognitive engagement^6,7^.

The performance of each AI model was evaluated based on the following criteria: accuracy of answers, reliability of answers, compatibility of difficulty categorization, correct answer rate by difficulty category, and correct answer rate by subtypes of questions. Analyses were performed using the chi-square test or Fisher's exact test. All statistical analyses were performed using the SPSS software package (version 25; IBM Corp., Armonk, NY, USA). The statistical significance of all tests was set at p≤0.05.

## RESULTS

The answers given by three different AI models were evaluated, and it was observed that ChatGPT-4 answered 74% of the questions correctly, Bard 66%, and ChatGPT 63.8%. It was found that the success rates decreased after asking, "Are you sure?" (66, 64.9, and 48.9%, respectively). There was no statistically significant difference between the three different AI models in the first response to the questions (p=0.254), but Bard (p=0.027) and ChatGPT-4 (p=0.018) were more successful than ChatGPT-3.5 in the second response to the questions. In the evaluation of the difficulty level of the questions determined by three different AI models, it was observed that Bard (p<0.001) categorized the questions more at medium difficulty compared to the other models (Table 1).

**Table 1:** Analysis of the answers of three different artificial intelligence models.

		ChatGPT-3.5 n (%)	Bard n (%)	ChatGPT-4 n (%)	p	p#
1st answer	Incorrect	34 (36.2%)	32 (34.0%)	24 (25.5%)	0.254	
	Correct	60 (63.8%)	62 (66%)	70 (74.5%)		
2nd answer	Incorrect	48 (51.1%)	33 (35.1%)	32 (34%)	**0.028**	**0.027** 1
	Correct	46 (48.9%)	61 (64.9%)	62 (66%)		**0.018** 2
Diffuculty of questions	Easy	47 (50%)	1 (1.1%)	33 (35.1%)	**<0.001**	**<0.001** 1
	Medium	45 (47.9%)	91 (96.8%)	57 (60.6%)		**<0.001** 3
	Hard	2 (2.1%)	2 (2.1%)	4 (4.3%)		

# Post-hoc analysis

1 between ChatGPT-3.5 and Bard

2 between ChatGPT-3.5 and ChatGPT-4

3 between ChatGPT-4 and Bard.

It was evaluated in terms of consistency of answer, and it was observed that ChatGPT-3.5 changed its answer in 66.7% of the questions. This rate was 32.2% in ChatGPT-4 and 1% in Bard. It was found that Bard changed answers to statistically significantly fewer questions than other AI models (p<0.001). The distribution of questions where ChatGPT-3.5 and 4 changed the answers was analyzed, and it was found that ChatGPT-3.5 changed answers from wrong to right in 20 questions, from wrong to wrong in 10 questions, and from right to wrong in 34 questions. In ChatGPT-4, these numbers were 10, 3, and 18, respectively.

In determining the difficulty distribution of the questions, it was found that Bard categorized the questions mostly as medium difficulty (p<0.001). Another important finding is that there was no significant difference in the difficulty distribution of the questions between the distribution made by physiatrists and ChatGPT-3.5 and ChatGPT-4.

The questions were categorized by the physiatrists into three categories: easy, medium, and difficult and the correct answer rates of the AI models were evaluated. In the intra-group evaluation, ChatGPT-3.5 answered 82.7% of the easy questions correctly and had a significantly higher accuracy rate than the medium-hard questions (p<0.001). For ChatGPT-4, this value was 82.7%, and a statistically significant difference was found (p=0.020). No statistically significant difference was found between question difficulty and correct answer rate in the intergroup analysis (Table 2). The questions were also divided into two different difficulty levels: low order and high order, according to Bloom's taxonomy method, and three different AI models were evaluated in terms of the correct answer rates of these questions. No significant difference was found within or between the groups.

**Table 2:** Assessment of initial artificial intelligence responses by difficulty level as determined by the authors.

	ChatGPT-3.5			Bard			ChatGPT-4			p
	Incorrect n (%)	Correct n (%)	p	Incorrect n (%)	Correct n (%)	p	Incorrect n (%)	Correct n (%)	p	
Easy	9 (17.3%)	43 (82.7%)	**<0.001**	13 (25%)	39 (75%)	0.062	9 (17.3%)	43 (82.7%)	**0.020**	0.525
Medium	18 (52.9%)	16 (47.1%)		14 (41.2%)	20 (58.8%)		10 (29.4%)	24 (70.6%)		0.143
Hard	7 (87.5%)	1 (12.5%)		5 (62.5%)	3 (37.5%)		5 (62.5%)	3 (37.5%)		0.446

The questions were categorized as specified by ABPMR, and the correct answers of different models were evaluated. It was found that ChatGPT-3.5 achieved 80.6% success in musculoskeletal system questions, while ChatGPT-4 achieved 85.2%, and Bard and ChatGPT-3.5 achieved 77.8% success in patient assessment and diagnosis. In addition, no statistically significant difference was found between different AI models in the question subheadings (Table 3).

**Table 3:** Comparison of artificial intelligence model performance by question categories as defined by ABPMR.

	ChatGPT-3.5		Bard		ChatGPT-4		p
	Incorrect n (%)	Correct n (%)	Incorrect n (%)	Correct n (%)	Incorrect n (%)	Correct n (%)	
Neurological disorders	11 (42.3%)	15 (57.7%)	10 (38.5%)	16 (61.5%)	6 (23.1%)	20 (76.9%)	0.304
Musculoskeletal medicine	6 (19.4%)	25 (80.6%)	11 (35.5%)	20 (64.5%)	8 (25.8%)	23 (74.2%)	0.354
Amputation	3 (60%)	2 (40%)	1 (20%)	4 (80%)	2 (40%)	3 (60%)	
Medical rehabilitation	3 (42.9%)	4 (57.1%)	2 (28.6%)	5 (71.4%)	1 (14.3%)	6 (85.7%)	
Rehabilitation problems	7 (46.7%)	8 (53.3%)	5 (33.3%)	10 (66.7%)	3 (20%)	12 (80%)	0.301
Basic sciences	4 (40%)	6 (60%)	3 (30%)	7 (70%)	4 (40%)	6 (60%)	
Patient evaluation and diagnosis	6 (22.2%)	**21 (77.8%)**	6 (22.2%)	**21 (77.8%)**	4 (14.8%)	**23 (85.2%)**	0.732
Electrodiagnosis	8 (72.7%)	3 (27.3%)	5 (45.5%)	6 (54.5%)	6 (54.5%)	5 (45.5%)	
Patient management	10 (32.3%)	21 (67.7%)	12 (38.7%)	19 (61.3%)	7 (22.6%)	24 (77.4%)	0.386
Equipment and assistive technology	2 (25%)	6 (75%)	3 (37.5%)	5 (62.5%)	3 (37.5%)	5 (62.5%)	
Applied sciences	8 (47.1%)	9 (52.9%)	6 (35.3%)	11 (64.7%)	4 (23.5%)	13 (76.5%)	0.357

## DISCUSSION

This study compares the performance of ChatGPT-3.5, ChatGPT-4, and Google Bard in the field of PMR, uncovering subtle differences in their abilities. All models performed similarly, but ChatGPT-4 led with a 74% success rate. Further testing showed ChatGPT-4 and Bard outperformed ChatGPT-3.5, especially in consistent answer quality, with Bard changing answers the least. The difficulty of questions as perceived by the ChatGPT closely matched expert opinions. Using Bloom's Taxonomy for question classification, all models showed similar performance across different cognitive demands. The study did reveal each model's strengths in patient assessment and diagnosis, with slight differences in specific areas.

In contrast to prior studies suggesting a clear superiority of ChatGPT-4 over its counterparts, our results present a more nuanced picture in the context of PMR-focused queries^3,8,9^. ChatGPT-4 indeed led the group with a 74% success rate, followed closely by Bard at 66%, and ChatGPT-3.5 at 63.8%, thereby not establishing a substantial margin of superiority for ChatGPT-4 as anticipated. When interpreting the results, it is notable that in a hypothetical examination with a passing threshold of 70%, ChatGPT-4 would have passed, potentially setting it apart from other AI models. However, this distinction, albeit statistically subtle, could be significant in practical terms. Yet, this interpretation is constrained by two pivotal factors. First, the ABPMR employs a unique scoring methodology, using scaled scores rather than raw percentages, which complicates direct comparisons. A study by Cuthbert and Simpson employed the United Kingdom and Ireland In-Training Examination (UKITE) as a stand-in for the Section 1 examination of the Fellowship of the Royal College of Surgeons (FCRS). The performance of ChatGPT was notably lower, at 35.8%, falling 30% short of the FCRS pass mark and 8.2% below the average human score. The authors attributed this shortfall to ChatGPT's limited capability for higher-order judgment and multilogical reasoning, essential for selecting the optimal answer in clinical scenarios. Their study highlighted a stark contrast between a 53% success rate in basic science versus a 0% in trauma, a disparity not observed in our research, even after categorizing questions and applying Bloom's taxonomy^10^. Isleem et al. focused on ChatGPT's performance on Orthopedic In-Training and Self-Assessment Examination questions from the American Academy of Orthopaedic Surgeons (AAOS)^11^. Out of 301 questions, ChatGPT correctly answered 183 (60.8%), hinting at varying performance levels across similar medical exams and possibly underscoring a lack of consistency in the model's medical proficiency.

Artificial intelligence is increasing its use in the field of medicine, as it is all over the world, and it affects healthcare in different ways. Today, AI is increasing its effectiveness in patient assessment, and the personalization of treatment plans, especially in areas such as radiology, pathology, and dermatology, thus creating an unprecedented change in patient care and medical practices^12-14^. The advantages of the use of AI systems in the field of health include the ability to predict potential health problems by analyzing individual health data, the recognition of diseases in the preclinical stage or early stage and the possibility of effective treatment, and the monitoring and care of the patient outside the hospital environment^15^.

In parallel with the increase in AI-mediated products used in the diagnosis, treatment, and follow-up of patients, regulatory rules are also being set. The concept of a medical device as software also encompasses AI-mediated products^16^. Therefore, to ensure patient safety and have certain standards, it must comply with the regulations of the medical device regulation. In addition, there are also ethical issues regarding the use of AI in the field of health. In this field, the guidelines published by different organizations, such as WHO and the European Union, also indicate increasing concerns and aim to create solutions^17^. Accordingly, there are still rules that need to be determined on vital points such as the openness of the algorithms used by AI technologies in decision-making, informing patients and obtaining informed consent, ensuring data confidentiality, and compliance with human rights and legal regulations^18^.

In parallel with technological developments, the term telerehabilitation is gaining importance in the field of PMR. In this period, when the elderly population and chronic diseases are increasing, the use of technological applications is gaining importance for the sustainability of health systems and public health. Studies have shown that the use of virtual reality systems in rehabilitation improves patients' quality of life, exercise compliance, and motivation^19,20^. In the near future, it will be possible to create patient assessment and therapy programs by combining virtual reality (VR) systems with AI systems. In this way, it will be possible to remotely assess the functional status of patients, create a personalized rehabilitation program, and remotely monitor their functional status.

While there are concerns about AI-mediated language models, there is growing evidence that they can be used in medical education. It is predicted that it will increase its weight in medical education due to its features such as enabling faster evaluation of students' written exam results and reducing the burden of instructors, thereby creating personalized learning suggestions and materials for students^21^.

The strengths of this study include the use of three different AI models and the first AI study on board-level questions in the field of PMR. However, this study has some limitations. The study lacks real-life data except for the authors' categorization of difficulty. Furthermore, this study used study questions from 2015 as the question set. It is suggested that future studies should be based on the use of real board questions and comparisons with real exam statistics.

## CONCLUSION

Overall, ChatGPT-4 achieved a 74% success rate in responding to PMR board-style questions, followed by Bard with 66% and ChatGPT-3.5 with 63.8%. The success rate of all three AI models was considered satisfactory. This shows that AI technologies, even in their current form, can solve complex clinical problems within the scope of PMR. Although it is predicted that AI systems will be used more by medical professionals in the future, it is recommended that the content suggested by AI should be carefully reviewed by medical professionals to reduce the risk of harm to patients.

## ETHICS APPROVAL

As this study involved no human or animal subjects and solely relied on AI-generated data, no ethical approval was required. However, the study was conducted in adherence to general ethical principles of research integrity and data confidentiality.

## Data Availability

The data associated with the paper are not publicly available but are available from the corresponding author on reasonable request.
